# More Satisfaction, Less Equality: Distributive Effects of Transparent Needs in a Laboratory Experiment

**DOI:** 10.1007/s11211-024-00434-0

**Published:** 2024-05-15

**Authors:** Bernhard Kittel, Sabine Neuhofer, Manuel C. Schwaninger

**Affiliations:** 1 Department of Economic Sociology, University of Vienna, Vienna, Austria; 2 Institute of Advanced Studies, Vienna, Austria; 3 YouGov, Zurich, Switzerland

**Keywords:** Distributive justice, Experimental research, Need-based justice, Social welfare, Transparency

## Abstract

Societies are confronted with the dilemma that need satisfaction requires transparent individual needs. We study the effect of information about others’ needs on the distribution of a joint endowment in a three-player network exchange game in a laboratory experiment. Need levels are exogenously given and either transparent (known to all three network members) or opaque (only known to the players themselves). The three players negotiate in dyads until two players agree on a distribution. We expect that the transparency of need thresholds raises need satisfaction but lowers equality. The results suggest that the members of the dyad who agree on the distribution can satisfy their own need thresholds even when information about thresholds is opaque. The effect of transparency on the remaining network member is antithetical: while transparency increases the rate of need satisfaction, it decreases the average share of allocations when needs are low. In the opaque condition, allocated shares are larger, but need satisfaction is lower. This reveals the ambivalent distributive effects of transparent need thresholds: Transparency helps those with the highest need thresholds, but it can hurt those with lower need thresholds, and it barely affects the ones with the most influence on the decision.

**Supplementary Information:**

The online version contains supplementary material available at 10.1007/s11211-024-00434-0.

## Introduction

Societies are confronted with distribution problems in manifold ways, which ultimately boil down to the problem of defining one or multiple criteria for the fair distribution of resources. Among these criteria, need-based justice constitutes one of the major principles of distributive justice (Traub & Kittel, 2020), which substantially influences the conception of social welfare (Esping-Andersen 1990, Sachweh 2016). This paper studies the distributive effects of transparent needs in light of a policy paradox: Any societal attempt to serve the principle of human dignity by providing support to human needs seems to inevitably violate dignity by the need to ascertain the legitimacy of need claims.

Conceptions of need-based justice start from an understanding of equality embedded in the idea of human dignity (Sen 1990; Nussbaum, 2011). Accordingly, there are socially acceptable minimum levels of various capabilities that are necessary for survival in human dignity in a particular society. To enable a dignified standard of living “all should get above a certain threshold level of combined capability” (Nussbaum, 2011, 24). Consequently, the need principle classifies a distribution of resources as just if it satisfies the need thresholds of all its members (Konow, 2001).

However, individual need thresholds depend on subjective criteria (Dean, 2010; Sen, 1990). Variations in moral values and intellectual capacities, physical skills, conceptions of the good, as well as tastes and preferences, inter alia, influence what people consider as their needs, which translates into different need thresholds and, hence, into different demands for resources. This heterogeneity implies that different people frequently have different need thresholds for the same capability. For example, people with a weaker immune system need more resources to recover their physical health after infection with a virus than people with a stronger immune system. These individual variations in need thresholds, together with the socially construed nature of needs, make the need principle a highly contested concept prone to different interpretations of what is needed in any particular context (Nelson, 2008).

The dependence on subjective information poses a serious challenge to the implementation of the principle of need-based justice. A distribution of resources that is legitimized by need is (a) subject to the potential overestimation of the recipient’s need, since the latter may have an incentive to ask for more than truly needed, and (b) subject to the potential underestimation of the recipient’s need, if the latter has no way of proving that the demand is indeed a need or is ashamed to express the need (Roosma et al., 2014). This uncertainty implies that others may transfer a larger or smaller share of the resources to the recipient than would be considered just according to the need principle if the situation could be properly assessed.

Ironically, while the transparency of need thresholds solves the underlying trust problem, it does so at the expense of the very fundament of the need principle: human dignity. Transparency implies publicly laying bare information about sensitive private issues. As the eminent social policy scholar Richard Titmuss (1968, 134) has remarked: “If all services are provided […] on a discriminatory, means-test basis, do we not foster both the sense of personal failure and the stigma of public burden?” In the same vein, Bo Rothstein (1998, 158) argued that this “very act of separating out the needy almost always stamps them as socially inferior.” Hence, the attempt to foster human dignity by implementing the need principle relies on a social mechanism that potentially undermines human dignity.

On the one hand, need-based justice has an intuitive appeal, can work as a compromise between an equal distribution and a performance-based distribution, can enable life in dignity for everyone, and may even have evolutionary advantages for group survival (Cronk & Aktipis, 2021). On the other hand, distributing resources according to the need principle can be a notoriously intricate exercise due to human diversity. Uncertainty about individual need thresholds can lead to trust problems and increasing transparency of need thresholds comes with social and administrative costs. Ultimately, it is a normative question whether human dignity is violated more seriously by an under-provision of resources or by the invasion of privacy through transparency along with the burden of social stigma, which every society must solve at least implicitly.

To identify the causal effect of transparency on the satisfaction of needs, we introduce an experimental design which varies the information on individual needs available to others. To the best of our knowledge, the causality entailed in the dilemma of transparent need thresholds has so far not been examined empirically. Yet, from a welfare policy perspective, it is crucial to understand the effects of transparency in distributive decisions. We thus aim to provide causal evidence regarding the distributional consequences of transparent versus opaque need thresholds. Contributing to the broader literature on social welfare states (Castles, 2010; Nelson et al., 2022; Van Oorschot et al., 2017), we offer an experimental base for informing and further developing the normative discourse on how social welfare regimes can deal with this policy problem.

To examine how reliable and objective information about individual, potentially heterogeneous, need thresholds affects the distribution of a limited resource, we focus on the smallest possible constitution of a society, a triad. We operationalize the triad as a three-line network (i.e. a partially connected triad) in a laboratory experiment. Thereby, we build on recent experimental research on need-based justice by Kittel et al. (2020), who find that transparent, heterogeneous need thresholds systematically affect the distribution of payoffs. However, they do not consider the critical problem that, in practice, individual need thresholds are rarely objectively verifiable by others, at least ex-ante. Here, we extend this design by varying the information available about others’ need thresholds between two treatments. Whereas in one treatment the need thresholds of all network members are transparent, that is, they are public information or “common knowledge”, they are opaque in the other treatment, that is, information about individual need thresholds is private.

To study need-based distribution outcomes, the three-line network has several favorable properties. First, the triad is the smallest group size in which socially emergent phenomena such as social embedding, norms, collective motivation, or status hierarchies develop (Lindenberg, 2015). Summarizing Simmel (1964), Yoon et al. (2013, 1457) argue that “triads tend to constrain emotions, reduce individuality, and generate behavioral convergences or uniformity because of ‘two against one’. Triads also allow for competition and coalition formation because individual fairness perceptions within dyads and across all network members may vary (Schwaninger, 2022). Furthermore, the three-line is a commonly observed social structure (Burt, 1992), which generates a power differential between network members (Skvoretz & Willer, 1993). After an agreement has been concluded between two network members, we can differentiate between the strong and weak agreeing network members in the dyad, and the third network member outside of the dyad. Importantly, power differences are particularly relevant for the identification and recognition of need thresholds due to the subjective nature of the necessary capabilities (Fraser, 1990). Finally, networks have been previously analyzed in the context of the need principle (Campennì et al., 2022; Hao et al., 2015) and allow us to compare our results to those of previous studies implementing the three-line network with (Kittel et al., 2020) and without need thresholds (Neuhofer et al., 2022; Schwaninger et al., 2019).

We find that transparent, in contrast to opaque, need thresholds raise the frequency of all network members’ need satisfaction. This effect is strongest for network members in weak structural positions and network members with high need thresholds. Transparent information is less significant for subjects in strong network positions and with low need thresholds. On the contrary, network members with low need thresholds tend to suffer from transparency because the network members who agree on a distribution use this information to target exactly the need threshold. When need thresholds are opaque, network members with low needs tend to obtain higher shares of the payoff because the equality principle is more prevalent. This reveals the ambivalent distributive effects of transparent need thresholds: Transparency helps those with the highest need thresholds, but it can hurt those with lower need thresholds if attention to need replace preferences for equality, and it barely affects the ones with the most influence on the decision.

The remainder of the paper is structured as follows. In Sect. "Transparency in Distributive Decisions", we provide an overview of the related literature. In Sect. "Hypotheses", we derive our hypotheses based on actors who are motivated by self-interest and their social value orientation. In Sect. "Experimental Design", we present the experimental design to test our hypotheses. In Sect. "Results" we report and discuss the results, and Sect. "Conclusion" provides a conclusion.

## Transparency in Distributive Decisions

### Transparency of Individual Conditions

Transparency is a highly debated topic in politics and societal processes alike, whereby most of the literature on transparency is focused on the macro and meso levels of society and targets the transparency of political and administrative decision-making processes (August & Osrecki, 2019, 2). Historically, the meaning of transparency has also been elaborated at the micro level of social interaction. In the tradition of Rousseau, the term refers to “the ‘honesty’ and immediate accessibility of someone’s ‘true’ beliefs and essence”, although “with the experiences of totalitarianism that infused Western societies with a horrific image of transparent individuals […] norms, practices, and structures of (inter-)personal observability are usually not framed in terms of transparency” (August & Osrecki, 2019, 23–24). Empirically, many countries have installed social welfare programs that involve an assessment of neediness and potential alternative income sources (Van Oorschot et al., 2017). However, people whose poverty is publicly exposed are often confronted with negative attitudes and experience stigmatization, shaming, and blaming (Walker, 2014), which suggests a potential downside of transparent needs.

At the micro level, transparency is thus a double-edged sword. On the one hand, it is exactly the availability of reliable information about the behavior of others and the intentions underlying this behavior that facilitates the development of mutual trust. Trust, in turn, fosters mutual solidarity and societal cohesion (Delhey et al., 2018). On the other hand, transparency can be a means of control and repression that undermines the development of trust, solidarity, and social cohesion at the individual, as well as the societal level. Trust is a substantial problem in welfare societies. Perceptions of welfare fraud and of underuse of benefits tend to undermine the legitimacy of welfare states (Roosma et al., 2016).

### Distributive Justice Principles

At the societal level, the distribution of resources is institutionalized in social welfare regimes. These constitute a normative order based on justice principles legitimizing the redistribution of resources. Social justice theory has identified four principles: equity, equality, entitlement, and need, which are considered appropriate for different social constellations (Mau 2004; Liebig & Sauer, 2016; Kittel, 2020; Nullmeier 2020) and welfare regimes (Sachweh 2016). Equity posits a strict proportionality between contributions and allocations. Equality prescribes allocating equal shares to all members of a community irrespective of previous contributions or individual attributes. Entitlement may be regarded as a consequence of the application of either equity or equality, but in social justice theory, it refers to some exogenous criterion or attribute such as custom or heredity (Hülle et al., 2018). The need principle, finally, demands that the allocated share depends on the need level. This implies that those who have more than the required threshold are expected to transfer resources to those whose endowment is below the threshold.

In the case of equity and equality, transparency is a matter of implementing simple procedures. The legitimacy of the application of these principles may be increased by public information on the rules and their correct implementation. Once the principles of equality or equity are established as a criterion for (re)distribution, the size of allocations is a matter of calculation and individual endowments become irrelevant. For entitlement, legitimacy may be divorced from transparency, as the general popular acceptance of the transfer of large public funds to royal families in Europe shows.

In the case of the need principle, the identification of the legitimate size of an allocation depends on private information held by those who claim to be in need. The severity of this problem grows when the need threshold is not objectively verifiable. For example, physicians should be able to determine the level of treatment the patient needs to recover. In most cases, however, it is difficult or even impossible to objectively identify individual need thresholds. Whenever others cannot differentiate between a lack of capabilities and a lack of effort it remains an issue for dispute whether a person needs more resources, for example, to learn, find a job, maintain health, ration food, or organize affordable housing, or whether they just want more resources to make life easier. In short, “[c]alculating the size of an equal share of something is generally much easier […] than determining how much a person needs of it to have enough” (Frankfurt 2015, 15). The difficulty of identifying needs makes this justice principle the most interesting and natural object for a study on the effects of transparency on resource distributions.

### Empirical Evidence on the Relevance of the Need Principle

Previous research has established that needs are a relevant criterion in distributive decisions. Information on the potential neediness of individuals appears to raise allocations. Subjects with higher needs receive larger shares of the resource in hypothetical decision situations (Bauer et al., 2022; Gaertner & Schokkaert, 2012; Konow, 2001; Yaari & Bar-Hillel, 1984). Water and food sharing occurs more frequently when recipients need water or are deprived of food (Brewis et al., 2019; Kause et al., 2018; van Dillen et al., 2021). Cues informing potential donors that recipients are “poor” raise monetary transfers (Brañas-Garza, 2006; Cappelen et al., 2013; Holm & Engseld, 2005; Smeet et al., 2015). Recent experimental evidence also suggests that resources are frequently allocated according to the need principle when homogenous (Cronk et al., 2019) and heterogenous (Kittel et al., 2020) needs are unambiguous and transparent. The latter study shows that subjects use randomly assigned needs, operationalized as a threshold for a specific aim, as focal points and distribute available resources accordingly.

Claessens et al. (2021) have experimentally studied the effect of the visibility of individual resources on need claims and need satisfaction in dyadic gift exchange relations. In this study, the authors focus on the uncertainty regarding the recipient’s endowment in long-term relationships. They find evidence supporting the importance of transparency for behavior: Recipients of potential benefits are greedier and potential donors are stingier in the opaque than in the transparent treatment, which suggests that transparency fosters cooperative behavior.

## Hypotheses

In the present study, we focus on the effect of transparency on the satisfaction of exogenous need thresholds. To﻿ study the difference between transparent and opaque need thresholds we have designed a laboratory experiment (see Sect. "Experimental Design" for the detailed description of the experiment). In this experiment, we examine networks with three members who must distribute a fixed amount of a resource among each other via majority rule, i.e. two of the three network members must agree. By design, subjects are allocated a need threshold, which must be satisfied in the distribution task to proceed to the next stage where they can earn money by solving a set of tasks. The capability to work is a commonly acknowledged need and most developed societies spend money, for example for training programs or language courses, on recipients in need to enable them to generate income by themselves. In the experiment, the need thresholds for the individual subjects vary, which means that some need more and others fewer resources to obtain the opportunity to work.

All network members know their own need threshold, but whether they know the need threshold of the other network members depends on the transparency (or opacity) of the thresholds. Treatments are either transparent, that is, all need thresholds within the network are common knowledge, or opaque, which means that all network members only know their own need threshold. When need thresholds are unknown to others, “needs-talk appears as a site of struggle where groups with unequal discursive (and non-discursive) resources compete to establish as hegemonic their respective interpretations of legitimate social needs” (Fraser, 1990, 164). This observation illustrates that power plays a crucial role when need thresholds are opaque. We thus implement the experiment in a three-line network, which provides one network member, the one in the center of the line, with structural power because this member can agree with one of the two other network members on a distribution, while the two network members on the periphery of the line cannot communicate with each other and, hence, can only agree on a distribution with the central network member.

Disregarding the transparency of need thresholds and assuming that subjects are purely self-interested, there is a clear game theoretic prediction in this situation. Theoretical models, subsumed under the umbrella of social exchange theory, build on rational choice theory to analyze networks. These models predict that the central network member will receive almost all payoff, while the agreeing, peripheral member receives a very small share of the payoff, and the network member outside the agreeing dyad is left empty-handed (Braun & Gautschi, 2006; Markovsky et al., 1988; Willer & Emanuelson, 2008). The reason is that the peripheral network members compete for inclusion in the agreeing dyad and prefer a small amount in the agreeing dyad over no payoff outside the agreeing dyad. Therefore, from a traditional rational choice perspective it makes no difference whether the other network members have needs or not, or whether the need thresholds are transparent or not. Hence, under the baseline assumption of self-interest, distributions should maximize the deciding dyad’s payoff.

In the three-line network without need thresholds, resources are more equally distributed within the agreeing dyad and, if this option is available (Schwaninger et al., 2019), the agreeing dyad frequently distributes a share of the payoff to the third network member (Neuhofer et al., 2022). When the distribution problem involves heterogeneous needs and need thresholds are transparent, agreements tend to adapt accordingly: they usually satisfy the need thresholds of the agreeing dyad and frequently also satisfy the need thresholds of third network members (Kittel et al., 2020). In these studies, the share allocated to the third network member increases with prosociality of the strong network member’s and, to a lesser extent, the weak agreeing network member’s prosociality (Schwaninger, 2022). These results imply that the satisfaction of others’ needs in a collective is more than the result of individual social value orientations; they reveal the presence of pro-social norms. Cialdini and Goldstein (2004, 597) argue that “relevant norms must be salient in order to elicit the proper norm-congruent behavior” when individuals attempt to “persuade others to engage in a particular behavior”. In this sense, negotiations about the distribution of resources in a triad entail implicit references to distribution norms: Both the descriptive dimension of norms (the orientation towards others’ behavior), and the injunctive dimension (mutual behavioral expectations), are implied in proposals, which signal own distribution preferences and behavioral expectations.

Building on the above results, we assume that distribution norms not only emerge in interaction, but also influence individual utility intrinsically (Krupka & Weber, 2013). In this vein, various models of human action include normative persuasions, which compete with fundamental self-interests in individual decision-making (Fehr & Schmidt, 2006; Lindenberg, 2013). Accordingly, narrow rational-choice models, which solely focus on self-regarding behavior, are now being replaced by wider conceptualizations (Opp, 2013), which posit that prosocial orientations are an important determinant of behavior. This human tendency is particularly important in the context of basic needs (Miller, 1999). At the same time, the literature identifies the equality principle as the default distribution norm in laboratory experiments in the absence of cues suggesting other principles (Diermeier & Morton, 2005; Selten, 1987).^1^ Equality addresses the immediate and decontextualized decision situation and can be operationalized as the size of the resource divided by the number of group members. In contrast, information about the needs of network members is a cue that subjects can use to choose the need principle as the appropriate distribution norm. Hence, when need thresholds are transparent, subjects may distribute resources either selfishly, according to the equality principle, or according to the need principle. How individuals weigh these factors depends on the decision makers’ social value orientation, which tells us how much individuals value their own payoff in comparison to applying a distribution norm that also takes others’ payoffs into account (Krupka & Weber, 2013).

Therefore, assuming that social value orientations are heterogeneously distributed among the population, individuals will prefer different distributions. Distribution norms guide how prosocial preferences are channeled and, therefore, more prosocial subjects are more likely to comply with distribution norms whereas more individualistic subjects are more likely to maximize their own payoff. Yet, the “selection of the norm to which one subscribes can also be explained by self-interest” (Elster, 1989, 115). This is crucial since complying with the equality principle or the need principle can have different distributional consequences depending on the distribution of needs. When the need thresholds of other network members are below the equal share, then the need-based distribution is “cheaper” than the equal distribution. Vice versa, when the need thresholds of others lie above the equal share, the need-based distribution is more expensive. We expect that the likelihood of network members to apply a more expensive distribution norm increases with their prosociality. When others’ need thresholds are below the equal distribution, more prosocial subjects are first more likely to satisfy others’ need thresholds and then to distribute the resources equally. When others’ need thresholds are above the equal distribution, they are first more likely to distribute resources equally and then to satisfy others’ need thresholds.

Absent verifiable information about others’ need thresholds, however, complying with the need principle is considerably more difficult. Transparent need thresholds generate objective focal points, which are vital to induce subjects to distribute payoffs according to their needs (Kittel, 2020). The lack of objective information generates uncertainty and distrust (August & Osrecki, 2019), which makes subjects greedier and stingier (Claessens et al., 2021). Therefore, we expect that subjects are more likely to disregard the potential need threshold of other network members. If the need thresholds are opaque to others but above the equal share, then both self-regarding and other-regarding subjects push toward a reduction of need satisfaction. Since more prosocial subjects prefer the equality principle when other cues are absent, they strengthen the downward preference of self-regarding subjects. If the need thresholds are below the equal share, individualistic and prosocial subjects pull into different directions. Since low need thresholds are likely satisfied irrespective of transparency and high thresholds are less likely satisfied in opacity, we expect need satisfaction to decrease when need thresholds are opaque to others.

### H1

Need thresholds are more likely to be satisfied when need thresholds are transparent than when they are opaque.

The strength of this effect is moderated by the position in the network, the individual influence on the decision, and the magnitude of the need thresholds. The share allocated to the weak network members, who have less bargaining power and, hence, influence over the distribution of payoffs, should be more negatively affected by opaque need thresholds. The effect should be strongest for the weak network member who does not make it into the agreeing dyad because the third network member is fully dependent on the members in the agreeing dyad. This is a consequence of the inherent nature of majority rule, which allows the majority to exploit the minority. Furthermore, network members with high need thresholds should be more severely affected by opaque need thresholds, because the probability that need thresholds are not satisfied by others increases with the size of the threshold. In sum, this reasoning leaves us with two additional hypotheses based on the network structure.

### H2

The negative effect of opaque need thresholds on the satisfaction of needs is stronger the lower the individual influence on the agreement is.

### H3

The negative effect of opaque need thresholds on the satisfaction of needs is stronger the higher the individual need threshold is.

As stated above, we expect these effects to operate at the level of negotiates and they should add to the effect of individual social value orientations. We thus include the latter as a control variable into the models presented below. According to Murphy et al (2011), an overwhelming majority of people are located on a scale between individualistic and prosocial orientations. When unambiguous, we use the term prosociality to refer to this scale. Previous work has shown that more prosocial individuals are more willing to transfer resources to those in need and that the structurally stronger actors’ social value orientations have a larger influence on the collective decision (Kittel et al. 2020).

## Experimental Design

We utilize the experimental threshold paradigm developed by Kittel et al. (2020) to study the relationship between the transparency of heterogeneous needs and need satisfaction. To examine the effect of information about individual need thresholds of network members on distributive outcomes, the experiment employs two between-subject treatments: In the transparent treatment, information about all need thresholds is publicly available. In the novel opaque treatment, information about need thresholds is private.

**Fig. 1:**
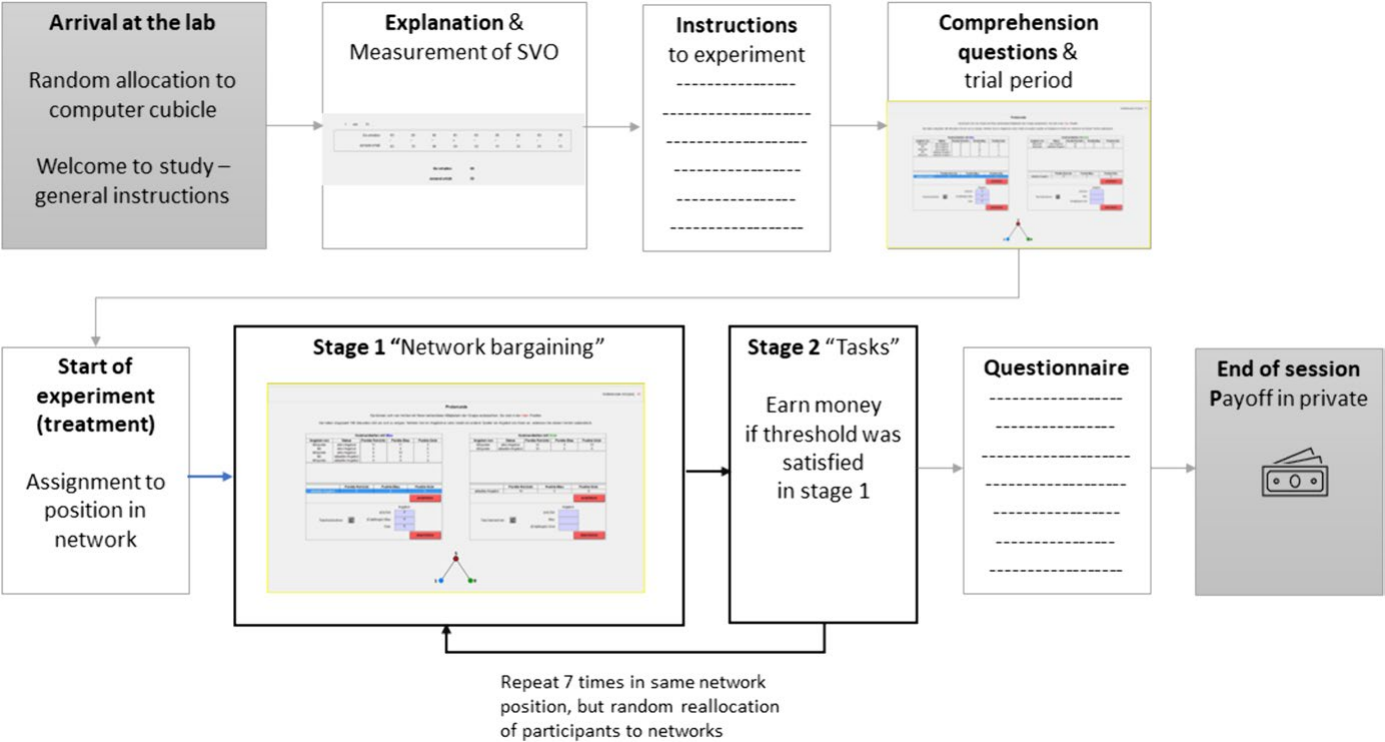
Flow Chart of the Experimental Design. *Note*: Participants received detailed instructions about the procedure of the experiment in text and with screenshots. For details, see the Appendix

Figure 1 displays the procedure and treatment variations of the experiment. In total, participants interacted in seven periods. Each period varies the need threshold levels. A period consists of two stages. In stage 1 (“dyadic network bargaining”, Sect. "Dyadic network Bargaining"), subjects bargain in dyads over the distribution of a fixed and exogenously given resource. In stage 2, subjects can generate additional payoffs by performing a set of real-effort tasks, if they have managed to satisfy their need thresholds (subSect. "Need Thresholds") in stage 1. Additionally, we measure social value orientations and justice attitudes (Sect. "Social Value Orientations and Justice Attitudes") to use as control variables in the regression analysis. Subjects were fully informed about the procedure of the experiment at the start of the experimental session. In appendix A (supplementary materials) we provide a detailed description of the experiment from the participants’ view.

### Dyadic Network Bargaining

24 subjects participate in each experimental session, equally divided into the transparent and the opaque treatments (12 subjects each). In total, participants play 7 periods: they proceed 7 times through stage 1 and 2.^2^ Prior to the first period participants (within treatments) are allocated to position A, B or C (Fig. 1). Subjects remain in their position (A, B or C) for the entire experiment, to avoid the development of reciprocity or insurance motives between subjects, that is, taking into account in one period of the experiment that one might need support from others in another period, and to obtain clear between-subject observations. However, in each period subjects are rematched randomly and anonymously into a new network, to avoid any development of personal reputation and longer-term partnerships.^3^

The three-line network implies that not all positions can send offers to each other. One subject (B) is connected to two other subjects (A and C), who are not connected, resulting in the network form A-B-C (Willer, 1999). Due to the structure of the network, bargaining position B is more powerful than A and C, since B is necessary to form a dyad. Therefore, we refer to subject B as the *strong network member* and to subjects A and C as the two *weak network members*. After an agreement has been concluded we can identify the two subjects that form the agreeing dyad based on their network position. In a dyad, subject B is the *strong dyad member*. The partner of subject B in the agreement, either subject A or C, is the *weak dyad member*. The remaining *third network member* is outside the agreement, but may be allocated a share of the endowment by the agreeing dyad.

In the first stage of each period, subjects bargain over the distribution of 24 points within their network. The points have two values for the participants. Firstly, they are converted into money and paid in cash to the subjects at the end of the experiment. Secondly, they determine whether the subject can earn additional income.^4^ The negotiations proceed in dyads (see Figure A “screenshot of negotiation page” in Appendix A). When a dyad agrees on a distribution of the 24 points amongst the three network members, this distribution is implemented.^5^ The connected network members (A and B; A and C) communicate by sending numerical distribution offers via the computer interface. In other words, subjects can propose and receive offers that are less than 24 points, whereby any distribution in non-negative integers is admissible, but they do not communicate verbally.^6^ Thus, subjects can emphasize their preferred distribution by repeating a particular proposal, but they cannot substantiate it verbally. In each period, subjects can send any number of offers and counteroffers, but only the latest offer from a participant can be accepted. The participants can send their offers simultaneously and freely whenever they see fit within the time limit (unstructured bargaining protocol). An agreement is reached when the recipient of a proposal accepts this offer by clicking on the “accept” button. Subjects must reach an agreement within the time limit of three minutes, or else the potential value created through exchange is lost and all three network members receive zero points. After an agreement has been concluded, all network members are informed about the bargaining outcome, and stage 1 ends.

### Need Thresholds

Need thresholds are represented by numerical thresholds assigned to every subject in each period. The thresholds indicate the minimal payoff share a participant needs from the network exchange game (stage 1) to receive payments from the real-effort task (stage 2). In the transparent treatment, the need thresholds of all subjects in the network are public information and displayed on the computer screen. In the opaque treatment, this information is private, and subjects can only see their own threshold displayed.

The distribution of the thresholds and their levels vary between periods. Table 1 displays the distribution in each period. The two otherwise identical experiments feature different threshold combinations.^7^ In experiment 1 the need thresholds vary across all network members and the sum of thresholds is held constant at 15 points in four of the seven periods. In two additional periods, the threshold of one weak network member is varied to create scenarios with more unequal threshold distributions. In experiment 2 the threshold of position B is held at a constant level of 5 points and the thresholds of positions A and C vary incrementally. In addition, experiment 1 contains one period where all thresholds equal 0; this period is excluded from analysis, as need satisfaction is always 100 percent. Experiment 2 contains one period where the sum of thresholds exceeds the available 24 points; this period is excluded from analysis, because not all needs can be satisfied by design, which implies that at least one threshold is never met.

**Table 1:** Need thresholds across positions and periods

	Experiment 1 (Vienna)						Experiment 2 (Hamburg)					
	Need thresholds						Need thresholds					
Period	A	B	C	∑	Diff	N (network)	A	B	C	∑	Diff	N (network)
1	0	5	9	14	9	32 + 32	1	5	1	7	4	32 + 32
2	9	1	5	15	8	32 + 32	9	5	5	19	4	32 + 32
3	1	5	12	18	11	32 + 32	5	5	12	22	7	32 + 32
4	5	9	1	15	8	32 + 32	5	5	1	11	4	32 + 32
5	0	0	0	0	0	32 + 32	12	5	12	29	7	32 + 32
6	9	5	1	15	8	32 + 32	9	5	9	23	4	32 + 32
7	5	5	5	15	0	32 + 32	5	5	5	15	0	32 + 32
Total number of network-level observations						224 + 224						224 + 224

In total, 384 subjects (192 subjects per city) participated, which equals 64 observations, i.e. 32 observations in treatment O and 32 in treatment T, on the network level per period in each city

In experiment 1, the need thresholds in period 5 are all equal to 0 and the thresholds are always satisfied. In period 5 of experiment 2, the sum of thresholds exceeds 24 points, and it is not possible to satisfy all need thresholds. These constellations are included in the experiment to explore further dynamics beyond the scope of this paper but excluded when calculating the need satisfaction rate in the following analyses

In the three-line network A–B–C positions A and C are considered “weak”; the central position B is considered “strong”

To summarize, the experiment varies the main treatment (transparent and opaque) between subjects and thresholds within subjects as an additional factor. The main treatment focuses on the difference between bargaining outcomes with and without information about the need thresholds of the other network members. The variation of need thresholds allows to test interaction effects between network positions and need thresholds.

### Social Value Orientations and Justice Attitudes

Before the bargaining experiment, we measured the participants’ social value orientations (Murphy et al., 2011). The SVO score is given by the angle on the circle segment in Murphy et al. (2011) measure, which ranges from competitive (− 16.26°) to altruistic (61.39°). We do not inform subjects about the results of this task until the very end of the experiment to minimize potential priming effects on the bargaining game.^8^ After the main experiment participants complete a questionnaire including order-related justice attitudes items from the Basic Social Justice Orientations (BSJO) scale (Hülle et al., 2018) and sociodemographic variables. We use support for the need principle to further validate decisions in the experiment^9^

### Measures

We focus on two outcomes: need satisfaction and payoff. Need satisfaction is a dichotomous variable that indicates whether the allocated sum is equal or larger than the individual threshold, which must be satisfied to earn additional points in stage 2. Payoff is a continuous variable which measures the points a participant receives in stage 1. The independent variables are a dichotomous measure of the transparency of others’ need thresholds (opaque or transparent) and the size of the threshold (number of points needed to proceed to the next stage). The control variables are the social value orientation, the period (a count variable running from 1 to 7 indicating the period in which the game is played), and the location (indicating the laboratory in which the experiment has be administered).

### Procedure

We conducted 16 sessions evenly weighted between all treatments, each consisting of 24 subjects, resulting in a sample of 384 subjects. As mentioned above, in each session half of the group was randomly allocated to the transparent and the other half to the opaque treatment. The experiment was programmed in z-Tree (Fischbacher, 2007). We ran experiment 1 in Vienna at the laboratory of the Vienna Center of Experimental Economics (VCEE) in November 2017. Since we exhausted the subject pool in Vienna, we ran a second set of sessions (referred to as experiment 2) at the WISO laboratory of the University of Hamburg in March 2018.^10^ The only change in the experimental protocol was the change of thresholds discussed in Sect. "Need Thresholds". An experimental session lasted about 100 minutes and the participants earned EUR 22.05 on average, ranging from EUR 8.00 to EUR 40.00.^11^

## Results

Altogether, 83.4 percent of all need thresholds are satisfied when they are transparent, and 78.2 percent of all need thresholds are satisfied when they are opaque. Relative to the outcome that we would observe if the members of the agreeing dyad were solely motivated by self-interest, namely, a need satisfaction rate of 66.7 percent, these results point to the presence of substantial other-regarding behavior in both treatments. The need satisfaction is 16.7 and 11.5 percentage points higher than predicted by the assumption of self-interest. Compared to opaque thresholds, the transparent need thresholds raise the average need satisfaction rate by 5.2 percentage points. A one-sided Mann–Whitney-U test on the session level, which is the independent unit of observation, supports H1 that need thresholds are more often satisfied when information is public than when it is private (*p* < 0.01). The effect is robust across experiment 1 (+ 4.8 percentage points, *p* = 0.01) and experiment 2 (+ 5.5 percentage points, *p* = 0.06). The following non-parametric and parametric analyses show that the influence of transparency on need satisfaction depends on the network position and the magnitude of the need thresholds.^12^

### Need Satisfaction, Transparency, and Need Thresholds

**Fig. 2:**
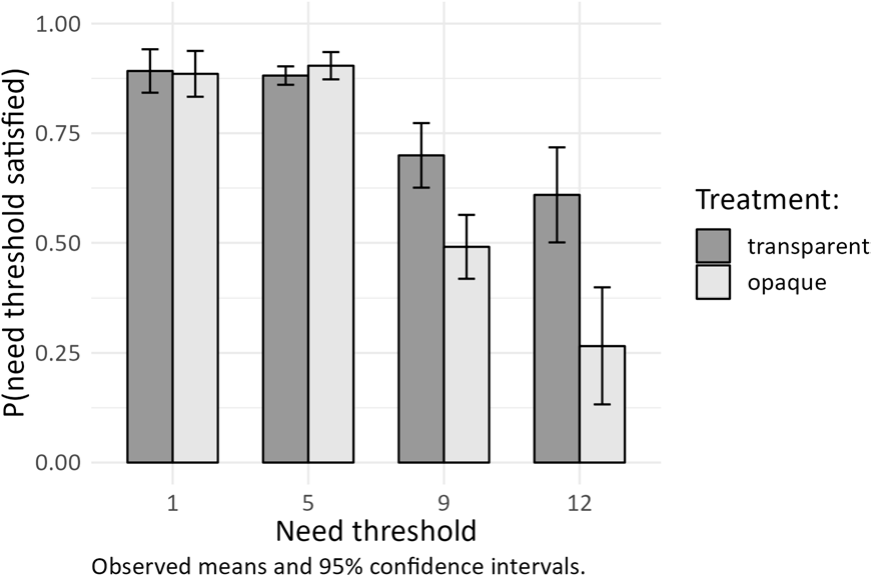
Need satisfaction across threshold levels. *Note*: The bars and confidence intervals refer to the share of satisfied needs for each threshold at the independent session level (*N* = 128, 32 sessions × 4 thresholds), aggregated from all individual bargaining outcomes (*N* = 2237); experiment 1 and 2 combined). Two periods are excluded from the analysis, due to the impossibility of complete need satisfaction or absence of need thresholds

Figure 2 shows the level of need satisfaction across the four implemented threshold levels. The individual need satisfaction rate decreases as the thresholds increase. The strongest decrease in the individual need satisfaction rate occurs when the threshold surpasses eight points, which amounts to an equal share of the available payoff. Individual need satisfaction decreases at an even steeper rate when information about the need thresholds is opaque. In comparison, there is a difference of 20.9 percentage points for a threshold of 9 (Mann–Whitney-U test, *p* < 0.01) and a difference of 34.4 percentage points for a threshold of 12 (Mann–Whitney-U test, *p* < 0.01).^13^ These results support H3 suggesting that the negative effect of opaque need thresholds is stronger for higher threshold levels. We only observe a difference between the transparent treatment and the opaque treatment when the individual need thresholds lie above the equal share of payoffs.

### Need Satisfaction, Transparency, and Network Position

**Fig. 3:**
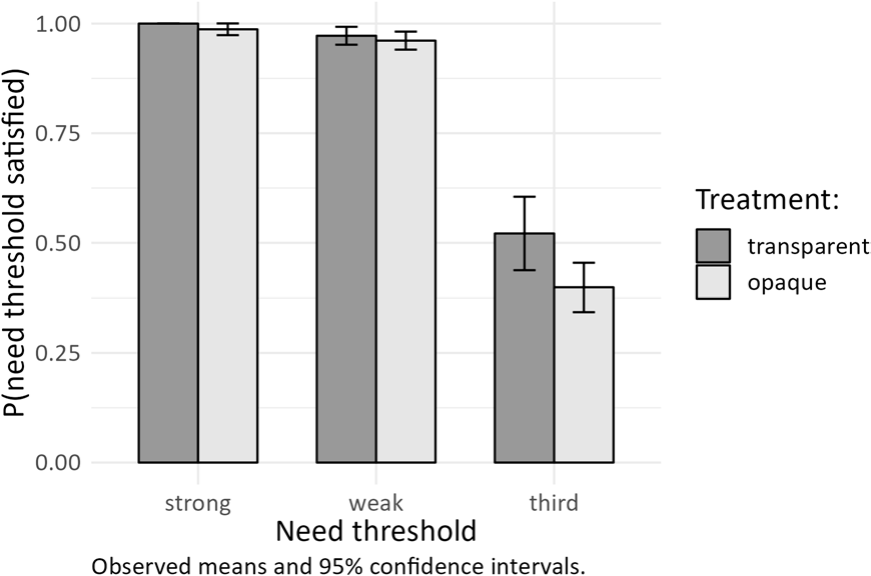
Need satisfaction across agreement positions. *Note*: The bars and confidence intervals refer to the share of satisfied needs for each threshold at the independent session level (*N* = 128, 32 sessions × 4 thresholds), aggregated from all individual bargaining outcomes (*N* = 2237); experiment 1 and 2 combined). Two periods are excluded from the analysis, due to impossibility of complete need satisfaction or absence of need thresholds

Figure 3 shows the variation of the need satisfaction rate across the network members after an agreement has been concluded. We can differentiate between the dyad that agrees on a distribution (one strong and one weak network position) and the third network member (weak position) who is outside the agreement. As predicted by the assumption of self-interest, the need satisfaction rate of third network members is significantly lower than for dyad members (Mann–Whitney-U test, *p* < 0.01). Additionally, the need satisfaction rate of third network members drops by 12.3 percentage points in the opaque treatment compared to the transparent treatment (Mann–Whitney-U test, *p* = 0.01). This result supports H2 that the effect of opaque need thresholds is stronger for network members with less influence on the agreement. Transparent need thresholds have a weaker effect on the need satisfaction rate of the dyad members. There is a statistically significant difference between the need satisfaction rates of strong network members in the different treatments (Mann–Whitney-U test, *p* = 0.01), but we can attribute this outcome to the invariable need satisfaction rate in the transparent treatment, in which the needs of strong network members are satisfied in every single agreement. Generally, subjects tend to only agree on a distribution of resources when it satisfies their own needs in both treatments.

Does this observation imply that subjects with higher needs are less likely to enter the dyad? In the transparent treatment, this is not the case. There is no significant influence of the need threshold on the frequency of dyad formation of subjects in the weak position (Wilcoxon test, *p* = 0.59). In the opaque treatment, this is also not the case if both thresholds lie below the equal split (Wilcoxon test, *p* = 0.85). However, subjects with lower need thresholds are significantly more likely to enter the dyad when the need threshold of the other network member lies above the equal split. In this case, the weak network member with the higher need threshold forms the dyad in only 39.8 percent of the observations, which is significantly less than 50 percent (Wilcoxon test, *p* = 0.04) and also significantly less likely than in the transparent treatment (Mann–Whitney-U test, *p* = 0.01). Thus, the treatment effect can partly be explained by the fact that subjects with lower thresholds have an advantage in the opaque treatment over subjects with higher thresholds.

### Accumulation of Disadvantages?

**Fig. 4:**
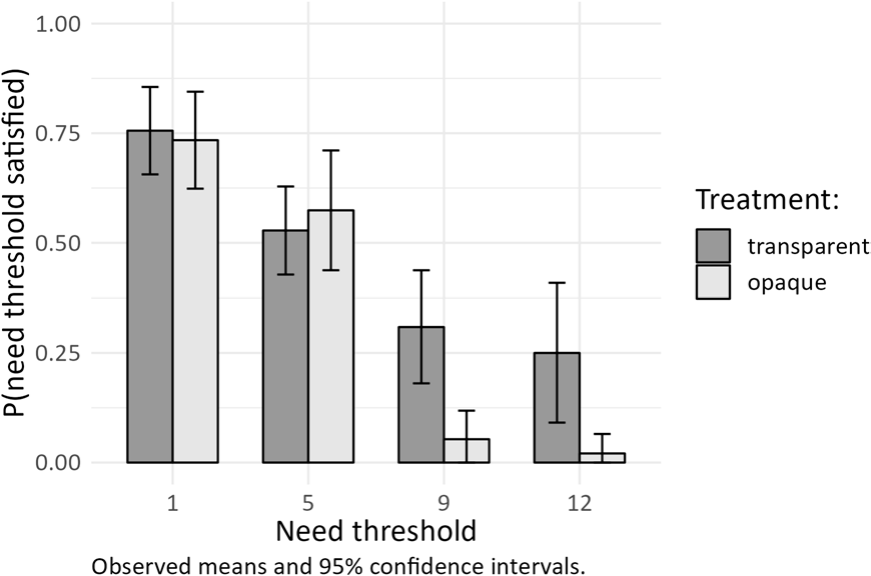
Need satisfaction of third network members. *Note*: The bars and confidence intervals refer to the share of satisfied needs for each threshold at the independent session level (*N* = 128, 32 sessions × 4 thresholds), aggregated from the third network member not in the dyad (*N* = 741); experiment 1 and 2 combined). Two periods are excluded from the analysis, due to impossibility of complete need satisfaction or absence of need thresholds (also see Kittel 2024).

Figure 4 shows the satisfaction of need thresholds across the four threshold levels of the third network members. The figure indicates that the need satisfaction of third network members also decreases significantly between the need threshold of 1 and 5 in the transparent treatment (Mann–Whitney-U test, *p* < 0.01) as well as the opaque treatment (Mann–Whitney-U test, *p* = 0.04), which Fig. 2 did not reveal. Furthermore, the results show that the agreeing dyad satisfies need thresholds above the equal share in more than 25 percent of the cases in the transparent treatment. However, in the opaque treatment, the dyad hardly ever satisfies the needs of the third network member when the need threshold exceeds the equal share of resources. Supporting H2 and H3, these results suggest that both structural disadvantages drive the differences between the transparent and opaque treatments.

### Determinants of Need Satisfaction of the Third Network Member

To further investigate the factors that influence the likelihood of the third network member’s need satisfaction, we estimate linear probability models.^14^ The dependent variable indicates whether the need threshold of the third member is satisfied or not. The independent variables include a treatment dummy for the opaque treatment, dummy variables for the magnitude of the need thresholds, and an interaction between the treatment and the need thresholds.^15^ The interaction tests H3 that the effect of transparent need thresholds increases with rising need thresholds. The control variables include the social value orientations of the network members to account for the fact that the outcomes can also be affected by individual differences in the propensity to comply with other-regarding distribution norms. We also control for the period of the network bargaining game and the location of the experiment. Finally, we cluster the standard errors on the independent group level to account for the interaction of the participants within a session.

Table 2 shows the results of the four specifications. Model I demonstrates that the likelihood of need satisfaction is significantly lower for the third network member in the opaque treatment. Likewise, the probability of the third network member’s need satisfaction increases (decreases) significantly when their need thresholds are low (high). In model II, we added the individual social value orientations as control variables. The likelihood of need satisfaction increases significantly for the third network member when the strong network member has a more prosocial orientation. The fact that the coefficients of the treatment and the threshold levels are hardly affected by the inclusion of social value orientations and remain statistically significant implies that these effects are not due to individual social value orientations but to a phenomenon that emerges at the group level. One participant’s prosocial or egoistic allocation proposal attains normative status in the deciding dyad, inducing the other participant to accept the proposal.

**Table 2:** Determinants of third network members’ individual need satisfaction

	DV: Need satisfaction of the third network member				
	I	II	III	IV	V
Opaque need thresholds (ref. = transparent)	− 0.124***	− 0.116**	− 0.116**	− 0.116**	− 0.139***
	(0.047)	(0.048)	(0.048)	(0.048)	(0.054)
Low need thresholds (ref. = Moderate)	0.279***	0.292***	0.304***	0.291***	0.184***
	(0.040)	(0.037)	(0.045)	(0.047)	(0.045)
High need thresholds (ref. = Moderate)	− 0.225***	− 0.211***	− 0.207***	− 0.220***	− 0.157***
	(0.035)	(0.037)	(0.037)	(0.040)	(0.058)
SVO of the strong dyad member		0.008***	0.008***	0.008***	0.008***
		(0.002)	(0.002)	(0.002)	(0.002)
SVO of the weak dyad member		0.001	0.001	0.002	0.002
		(0.002)	(0.002)	(0.001)	(0.001)
SVO of the third network member		− 0.002*	− 0.002*	− 0.002	− 0.002
		(0.001)	(0.001)	(0.002)	(0.002)
Period			0.008	0.008	0.008
			(0.011)	(0.011)	(0.011)
Location: Vienna (ref. = Hamburg)				− 0.052	− 0.051
				(0.054)	(0.054)
Opaque x low need thresholds					0.214***
					(0.069)
Opaque x high need thresholds					− 0.125*
					(0.068)
Constant	0.507***	0.354***	0.325***	0.332***	0.344***
	(0.038)	(0.071)	(0.076)	(0.080)	(0.077)
Observations	741	741	741	741	741
Adjusted R^2^	0.145	0.198	0.198	0.199	0.212

Linear probability models with clustered standard errors on the independent session level

^***^ *p* < .01, ** *p* < .05, * *p* < .10

Need satisfaction is a dichotomous variable indicating whether the individual need threshold of the third network member not in the dyad is satisfied

*Low need thresholds* include the combinations 1–5-1, 5–5–1, 5–9–1

*Moderate need thresholds* include the combinations 5–5-5, 9–5-0, 9–5–1, 9–1-5, 9–5–5

*High need thresholds* include the combinations 9–5-9, 12–5–1, and 12–5–5. The first and third numbers represent the need thresholds of weak network members the second numbers those of strong network members, respectively (see, Table 1)

Period 5 of both experiments are excluded from analysis, due to non-satisfiability or absence of need thresholds

In models III and IV, we control for period effects and location of the experiment, which have no significant impact on need satisfaction. Finally, in model V, we include an interaction term between the opaque treatment and the need thresholds. The results imply that opaque need thresholds affect the need satisfaction rate of the third network member especially when this member’s need threshold is high.^16^

### Transparent Needs and the Payoff of the Third Network Member

Do third network members earn more when their need thresholds are transparent? When we add the payoffs from the network bargaining game and the real-effort task, there are no significant differences between the two treatments (Mann–Whitney-U test, *p* = 0.65). This is surprising. Since the need thresholds of third network members are more often satisfied in the transparent treatment, third network members earn on average more payoff from the real-effort tasks in the transparent than in the opaque treatment (Mann–Whitney-U test, *p* < 0.01). Hence, we would also expect third network members to obtain higher profits in the transparent treatment than in the opaque treatment across the entire experiment, which is not the case.

This observation demands an explanation. It turns out that third network members receive higher payoff shares from the dyad in the opaque treatment than in transparent treatment (Mann–Whitney-U test, *p* = 0.09). The regression results in Table 3, where we estimate the same models as in Table 2 but with the third network member’s payoff as the dependent variable, corroborate the non-parametric test results. The influence of opaque need thresholds on the payoff of the third network member is statistically rather weak, but strikingly, the influence is positive (model II to V), whereas the influence on the need satisfaction is significantly negative. This means that when need thresholds are opaque, the payoffs allocated to third network members increase. The effect is strongest when the need thresholds are low (model V). Beyond that, the third network member’s payoff increases (decreases) when the total need thresholds in the network are low (high) because more (fewer) resources are available after satisfying the need thresholds. Further, the results reveal that the social value orientation of the strong and weak network members in the agreeing dyad significantly affect the payoff of the third network member. In comparison, the social value orientation of the agreeing weak network member has no significant influence on the need satisfaction of the third network member, as shown in Table 2. Also, over several periods, the agreeing dyad allocates significantly less to third network members, whereas the need satisfaction rate does not decline. Hence, inequality increases over time. This might be interpreted as an indication that when individuals learn to utilize their structural advantage better, the normative force of the equality principle decreases, whereas the normative force of the need principle remains stable.

**Table 3:** Determinants of third network members’ payoff (level of analysis: individual)

	DV: Payoff of the third network member				
	I	II	III	IV	V
Opaque need thresholds (ref. = transparent)	0.705	0.798*	0.799*	0.802*	0.795*
	(0.470)	(0.480)	(0.480)	(0.468)	(0.479)
Low need thresholds (ref. = Moderate)	0.453***	0.570***	0.415***	0.292*	0.012
	(0.176)	(0.161)	(0.158)	(0.156)	(0.204)
High need thresholds (ref. = Moderate)	− 1.063***	− 0.798***	− 0.781***	− 1.088***	− 0.822**
	(0.260)	(0.265)	(0.261)	(0.254)	(0.362)
SVO of the strong dyad member		0.072***	0.072***	0.077***	0.077***
		(0.014)	(0.014)	(0.014)	(0.014)
SVO of the weak dyad member		0.029**	0.028**	0.038***	0.038***
		(0.014)	(0.014)	(0.013)	(0.013)
SVO of the third group member		− 0.006	− 0.006	0.002	0.002
		(0.012)	(0.012)	(0.012)	(0.012)
Period			− 0.233***	− 0.231***	− 0.231***
			(0.049)	(0.048)	(0.048)
Location: Vienna (ref. = Hamburg)				− 0.926*	− 0.926*
				(0.496)	(0.496)
Opaque x low need thresholds					0.560*
					(0.319)
Opaque x high need thresholds					− 0.532
					(0.497)
Constant	4.132***	1.995***	2.730***	2.879***	2.881***
	(0.308)	(0.604)	(0.636)	(0.665)	(0.648)
Observations	894	894	894	894	894
Adjusted R^2^	0.033	0.128	0.144	0.154	0.156

Maximum likelihood models with clustered standard errors on independent session level

^***^ *p* < .01, ** *p* < .05, * *p* < .10

Payoff is a continuous variable measuring the number of points allocated to the third network member not in the dyad

*Low need thresholds* include the combinations 0–0–0, 1–5-1, 5–5–1, 5–9–1

*Moderate need thresholds* include the combinations 5–5–5, 9–5–0, 9–5–1, 9–1–5, 9–5–5

*High need thresholds* include the combinations 9–5–9, 12–5–1, 12–5–5, and 12–5–12. The first and third numbers represent the need thresholds of weak network members the second numbers those of strong network members, respectively (see, Table 1)

Period 5 of experiment 2 is excluded from analysis due to non-satisfiability of all three thresholds

Hence, when need thresholds are low and transparent, the dyad members tend to use this information to implement lower allocations to the third network member. In the opaque treatment, the dyad is more likely to agree on an equal distribution, which increases the average payoffs of the third network member. In sum, the third network members jointly earn less from the real-effort task but more from the bargaining game in the opaque treatment, which overall equalizes the average payoff compared to the transparent treatment.

## Conclusion

In this paper, we have studied the effect of transparent need thresholds on the prevalence of the need principle in a controlled environment. We find that transparent need thresholds lead to more need satisfaction but less equal distributive outcomes. Overall, the results of this laboratory experiment suggest that the transparency of need thresholds significantly increases their satisfaction. The need threshold appears to legitimize distributions that depart from the equal split of resources. Notably, when need thresholds are higher than the equal split, the likelihood of third network members’ need satisfaction increases when their need thresholds are transparent, i.e. verifiable by “objective” information. In contrast, when unverifiable need thresholds are low, the even split is more attractive for agreeing dyads with more prosocial orientations, thus resulting in higher payoffs for the third network member. Together, these results suggest that the effect of transparency is ambivalent: When high need thresholds are transparent, the chance that others will satisfy them increases; however, when low needs are transparent, others can also use them to legitimize unequal allocations of resources in a self-serving way. The results provide empirical evidence that the need principle can override the equality principle and, thus, not only be used benignly to motivate need-based distributions but may also be instrumentalized by decision-makers to legitimize self-serving distributions. Given that these effects are present while social value orientations are controlled, they cannot be explained by the latter but indicate that the satisfaction of others’ needs obtains normative force in the negotiation.

The empirical evidence presented in this paper speaks to two important questions. First, the verifiability of need thresholds appears to be an important condition for a successful distribution of resources based on need. Clear and verifiable (objective) information about the state of a person’s needs strongly raises the probability that those needs are satisfied. Hence, the evidence suggests that having full information about each citizen’s need thresholds would be efficient to avoid wasting resources and increase the chance of supporting all those in need. In practice, opaque need thresholds are arguably more frequent than naturally transparent ones. Therefore, from a social policy perspective, it would be helpful to enable decision-makers to assess individual need thresholds. However, the principle of human dignity calls for a restraint in institutionalized control. This points to a policy dilemma. On the one hand, transparency of need thresholds improves need satisfaction and enables a life in dignity. On the other hand, it might stigmatize recipients and interfere with the idea of a dignified life. Other factors such as trust and social cohesion may alleviate this need for verifiable information. If people can trust in others’ solidarity or believe in others’ cooperative orientations, such that only those in need apply for social benefits, resources should more likely be redistributed to the truly needy without the public exposition of their need. Further experimental research might test these alternative social mechanisms.

Second, self-serving decision-makers can refer to low needs to legitimize low transfers to structurally weak group members. An illustration of this phenomenon can be found in the coalition agreement of the Austrian government that took office in December 2017: The governing parties agreed to change the regulation of child benefits for children living abroad whose parents work in Austria, which eventually tied the level of benefits to the cost of living index in the child’s country of residence. Essentially, the idea was to reduce benefits for children who live in a country with lower living expenses. The underlying logic is that these parents need fewer benefits than parents with children living in Austria because of the lower cost of living. The policy was declared “a step toward more justice” by the Austrian chancellor at the time. However, it turned out that such practices are highly controversial. In 2022 the Court of Justice of the European Union declared this policy illegal.^17^

In sum, “[p]ublic information has attributes that make it a double-edged instrument. On the one hand, it conveys information on the underlying fundamentals, but it also serves as a focal point for the beliefs of the group as a whole” (Morris & Shin, 2002, 1521). Our results suggest that the tradeoff inherent in making needs transparent might be best decided by reference to the need levels reported by citizens. When the individual level of need satisfaction is high, then the benefits of identifying the need of a recipient might outweigh the individual cost of intruding into their privacy. At least in our study, subjects with high need thresholds benefit considerably from transparent needs. However, when the suspected individual level of need is low, it might make less sense for people to give up their privacy because, on top of privacy issues, the information about neediness may be utilized to their disadvantage to justify allocations that leave them at the edge of need satisfaction but worse off than others.

Naturally, our findings also come with certain limitations. First, we have studied need-based justice in a three-line network. We have focused on this decision structure in a controlled environment due to the numerous favorable properties of experiments. Focusing on one specific social structure, or on a structure itself as opposed to a voting mechanism, for example, always goes hand in hand with concerns regarding the generalizability of the results. Further developments and replications should address other power structures, larger networks, and other decision modes. In addition, two particularly relevant questions with respect to policy implications of laboratory experimental research relate to the quality of information and personal characteristics of the needy. Different levels of uncertainty in the available signal about need thresholds would allow to elaborate the effect of transparency on need satisfaction in a more fine-grained way. This effect may interact with the effect of individual responsibility for the need, which is usually operationalized in laboratory experiments by the level of risk-taking (e.g., Esarey et al., 2012).

Moreover, whereas a laboratory experiment has allowed us to derive causal evidence on the effects of transparent need thresholds, the findings from it should be complemented by other research designs. For instance, field, survey, or interview studies allow researchers to explore whether the identified mechanisms change in the context of different kinds of needs. Lastly, while the purpose of this study was to identify the distributional effects of transparent and opaque need thresholds conditional on the network position and the magnitude of the need thresholds, we were less concerned about the psychological drivers of the effects. Future work could disentangle whether social trust, lack of knowledge about the possible need thresholds, or effects on the self-image explain the influence of transparent need thresholds on distribution outcomes.

### Supplementary Information

Below is the link to the electronic supplementary material.

Supplementary file1 (PDF 1211 KB)

## Footnotes

1 Another factor causing groups to select the equal distribution is the requirement of unanimity, which allows all members to veto a decision allocating less than an equal share to them (Miller & Vanberg 2013). They also find that participants learn quickly to adapt to a change in the decision context.

2 Prior to the first period, all subjects participate in a practice period that is not relevant for payoffs to familiarize themselves with the setup.

3 For example, a subject allocated to position A will remain in position A for all 7 periods. However, subjects cannot identify each other. In each period a subject in position A will be matched with a subject in position B and a subject in position C. Thus, they have to assume that they play with different people in each new period.

4 We use standard tasks for generating additional income in the laboratory experiments, such as math problems, trivia questions and simple counting of displayed objects. Stage 2 is relevant for the total payoff when the need threshold in stage 1 of the period selected for the payoff is satisfied.

5 See the instructions in Appendix C in the Supplementary Materials for the description of this stage as it was presented to participants.

6 We prevent other forms of communication to control for the influence of cheap talk on the agreements.

7 We do not randomize the sequence of the threshold combinations between the sessions since the number of sessions would have been insufficient for a successful randomization, and instead choose a constant sequence.

8 An alternative would be to administer the social value orientations measure at the end of the experiment. However, this sequence can influence the results of the measure significantly, while the influence of an other-regarding measure on the bargaining outcomes remains insignificant when the experiment reveals the outcome of the measures only at the end of the experiment (Schwaninger, 2022).

9 Details are provided in Appendix B in the Supplementary Materials.

10 Table A1 in the Supplementary Materials provides an overview of the demographic data of the subjects in the sample. VCEE uses ORSEE (Greiner, 2015) and the WISO laboratory uses hroot (Bock et al., 2014) to recruit participants.

11 At the end of each experimental session, one out of the seven periods was selected randomly for payoff and added to a random draw from the social value orientation measurement.

12 Tables A2-A7 in the Supplementary Materials show the results broken down for each scenario.

13 All tests remain robust when we do not aggregate the data on the independent group level and perform tests of equal proportions on the individual level.

14 We also calculated logit regression models, which we display in Table A8 in the Supplementary Materials as robustness tests. The results are robust, but as pointed out by an anonymous reviewer, the interpretation of the coefficients from logit models is not straightforward when including interaction effects (Ai & Norton, 2003).

15 Here, we grouped the need thresholds within the network into low, moderate and high need thresholds to make the results more assessable. The results are robust if we control for the threshold levels via individual dummy variables, as displayed in Table A9 of the Supplementary Materials.

16 The regression results suggest that strong network members indeed have a dominant influence on the distribution of resources. To cross-validate our results, we use this information to compare the behavior in the bargaining game with a survey measure of subjects’ support for the need principle (see Appendix B in the Supplementary Materials).

17 See Government of Austria (2017, 100), Die Presse, 4 January 2018, https://diepresse.com/home/innenpolitik/5347733/Schritt-zuGerechtigkeit_Regierung-kuerzt-Familienbeihilfe-fuer, Der Standard, 16 June 2022, https://www.derstandard.at/story/2000136616024/eugh-indexierung-der-familienbeihilfe-in-oesterreich-rechtswidrig?ref=article.
