# Supplementary material for: More Satisfaction, Less Equality: Distributive Effects of Transparent Needs in a Laboratory Experiment
Source: Soc Justice Res. 2024 May 15;37(2):122–48. doi: 10.1007/s11211-024-00434-0 (PMC11161535; doi:10.1007/s11211-024-00434-0)
Supplement: Supplementary file 1 — Supplementary file1 (PDF 1211 KB) [file 11211_2024_434_MOESM1_ESM.pdf]

More Satisfaction, Less Equality:
Distributive Effects of Transparent Needs
in a Laboratory Experiment

Bernhard Kittel, Sabine Neuhofer & Manuel C. Schwaninger

Supplementary Materials

Appendix A. The experimental procedure in detail, from the perspective of participants

Students of universities located in Hamburg and Vienna can register at the experimental laboratory to be invited to participate in experimental studies. Once a researcher starts data collection, registered students are invited by mail. They do not know what the study is about, but they know that they will be compensated financially, depending on their own and other participants' decisions.

Figure 2 depicts a flowchart of the experimental procedure. Once participants arrive at the laboratory, they randomly draw a numbered card and take their seat at the corresponding PC. All PCs are separated by cardboard walls to ensure privacy of decisions. Then the laboratory staff read aloud the code of conduct of the laboratory and hands out a printed version of the instructions, which are then read aloud by one staff member.

In the present study, participants are informed that the experiment has two parts. In the first part we measure social value orientations (see Instructions page 2 in Online Supplementary Material); participants complete the task, which entails six choice sets dividing money between themselves and a randomly drawn person in the room. Then, the instructions of the second part of the experimental study are read aloud and participants answer comprehension questions concerning the instructions. Participants are invited to raise their hands if they want to ask a question. Once all questions are completed correctly the experimental session starts. Participants are randomly allocated to networks and network positions with a specific need threshold by the computer program. This information is displayed on the screen. In a "practice period", participants are familiarized with the task and the input mechanism.

Figure A: Screenshot of stage 1 "dyadic network bargaining"

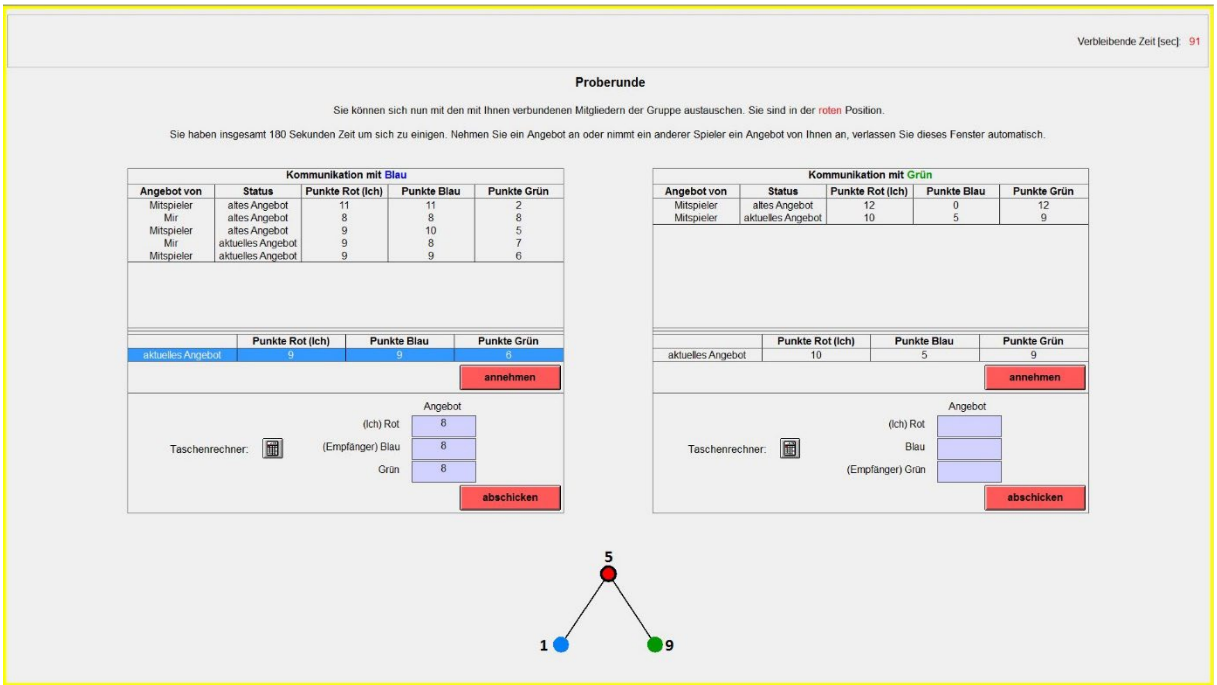

Note: The screenshot shows the perspective from the perspective of the central (strong) network position B (referred to as "red position" in the experiment) in the transparent treatment.

Stage 1: Figure A "Screenshot of stage 1" shows the computer screen from the perspective of the red (central) position. Positions blue and green only see one half of the screen (communication with the red position). The bottom of the screen displays the network: the position of the participant who sees this screen is highlighted with a black circle (red in the example) and the numbers next to the colored

dots indicate the need threshold (changes between periods). In the example, the red position has a threshold of 5 point and sees (transparent treatment) that the blue position has a threshold of 1 point and the green position has a threshold of 9 points. The opaque treatment differs from the transparent treatment by omitting he information on the thresholds of the other participants.

Communication with other network members and finding an agreement about the distribution of points: the participant holding a red position can send an offer to the blue position (communication box, titled "Kommunikation mit Blau", on the lefthand side of the screen, or the green position on the righthand side) by typing the proposed amounts in the three light blue boxes and clicking on the red submit button ("Abschicken"), which sends the offer to the blue position. It then appears in the dialog box above on the screen of the sender (red position) and recipient (blue position). Each participant can send multiple offers in their communication channels; these offers are presented in a list. The latest received offer is displayed in the middle of the communication box. To accept an offer, the participant clicks on the offer and once it is selected (indicated by turning bright blue) s/he clicks on the red button next to it to accept it ("Annehmen"). The first offer that is accepted in the network is implemented. Network members must reach in agreement within 3 minutes otherwise the resource dissipates.

Stage 2: the agreement is displayed on the screen and participants are informed whether or not the distribution satisfies their needs. Subsequently, participants can solve tasks and earn money with each solved task. If they did not satisfy their threshold, participants can solve tasks but do not earn money with it. At the time limit of the task solving stage, participants are informed about their earnings. Then participants start a new period.

In total, seven periods are played. In each new period participants remain in the network position of the first period, but they are randomly matched with different participants in each new round. Thresholds, which were chosen for theoretical reasons and experiences with previous experiments, vary across rounds. After the seventh period participants complete a short questionnaire before they are informed about their earnings. One period from the first part and one period of the second part of the experiment are randomly selected. Subsequently, participants are called sequentially to the staff desk where they are paid out the amount corresponding to their card. This procedure ensures anonymity. Afterwards they leave the laboratory.

Appendix B. Validation of support for the need principle

To cross-validate our results, we use this information to compare the behavior in the bargaining game with a survey measure of subjects' support for the need principle. For each strong network member, we calculate the share of agreements that satisfies the need thresholds of all network members. The correlation between the share of need thresholds that strong members satisfy and their self-reported attitudes toward the need principle is 0.28 ($p = 0.02$) for the transparent treatment and 0.18 ($p = 0.15$) for the opaque treatment. This means that subjects who are more likely to satisfy need thresholds in the bargaining game are also more likely to support the need principle in the post-experimental survey. However, the correlation is only significant when the need thresholds are transparent, which aligns with our argument that knowledge about the need thresholds of others is a prerequisite for the application of the need principle.

Appendix C: Experimental instructions translated from German

Welcome! Along with the other participants you will make several decisions today with the opportunity to earn money. These instructions explain the course of the experiment. It is important that you read the instructions carefully so as to gain a thorough understanding of the situations in which you will make decisions.

In case any instructions seem unclear while reading or if you have any other questions, please raise your hand. We will then come to you to answer your questions individually**. Please do not ask your questions openly. Please do not talk with other participants and do not pass any information to others. Complying with these rules is very important for the scientific value of the experiment.**

The anonymity of all the participants will be maintained throughout the entire experiment. The amount you earn will depend on your decisions and the decisions of other participants. Throughout the experiment, we do not speak of Euros but of **Points**. After the experiment, these will be converted at the following exchange rate:

1 Point = 1,1 Euro

The experiment will last about 2 hours. Take your time to decide. In some situations, short waiting times can occur, because the program waits for all participants to proceed. At the end of the experiment, you will be paid individually, privately and in cash.

Experiment 1

In this task you will be randomly paired with another person and you have to decide how you want to allocate certain numbers of Points between yourself and this other person. In the following we will refer to the other person as "Other". You do not know who the Other is and the other does not know who you are. All your decisions remain completely confidential and anonymous. For each of the following cases, please indicate the distribution you prefer the most.

Your decisions will generate money for both yourself and the other person. In the example below, a person has chosen a distribution which pays 2.16 Points to her- or himself, while the other person receives 1.41 Points.

Example

You receive	1.50	1.63	1.76	1.89	2.03	2.16	2.29	2.42	2.55
Other receives	3.00	2.68	2.36	2.04	1.73	1.41	1.09	0.77	0.45

There are no right or wrong answers in this task. This task is only about which option you personally prefer. After you have made your decision, mark the respective position along the midline. You can

only make one mark for each case. As you can see, your choices will influence both the number of Points you receive and the number of Points the Other receives.

In total all participants make 15 decisions of this kind. At the end of the experiment one decision will be drawn randomly and paid to you and the other person. This means that you will receive Points for exactly one of your decisions and you will receive money from exactly one decision of another participant.

Once all participants have finished Experiment 1, we will hand out the instructions for Experiment 2.

Experiment 2

For this experiment you will be matched into **groups of three** in each period. The experiment has **seven periods** in total**.** At the start, you are assigned to a position in a network. You will remain in this position for the entire experiment (labelled with colors red, green, and blue). However, in each period you are matched into a new group of three (i.e. with two new group members). Also, in each period a **threshold** is randomly assigned to you. This applies to all participants equally.

Each period has two parts (Part A and B). In Part A, you can communicate with the members of your group to find an agreement about how to allocate 24 Points. If you obtain enough Points to reach the threshold that has been randomly assigned to you, you can earn additional Points in Part B.

Part A: Allocation of Points

In each period of Part A, you can exchange offers with the other group members to **allocate 24 Points**. Depending on how you are connected to your group members, you will see either one or two windows on your computer screen. In each of these windows you can separately exchange offers with one other person. You will receive information about all thresholds in each period. The thresholds that has been assigned to you and the other group members are displayed as numbers next to the colored circles that mark the positions. [*Opaque treatment*: You will receive information about your threshold in each period. The thresholds that has been assigned to you is displayed as a number next to the colored circles that marks your position.]

You can communicate using the windows on your screen, in which you can send **offers to allocate the 24 Points**. You can send and receive offers from the players you are connected to (see example in the figure below). After **thirty seconds** you can accept offers. You can only accept the most recent offer. You have a time limit of **three minutes** to find an agreement. If no pair agrees on an allocation, no group member receives any Points. There can be **only one agreement** per period within the three-person group. If the two other group members agree on an allocation, this allocation is also binding for you even if you were not part of the agreement. Hence, the first agreement concluded between two players is valid for all group members.

If you obtain enough Points to reach the threshold that has been assigned to you, you can earn additional Points in Part B. The thresholds are randomly allocated each period among the group and

can change each period. The threshold of each player is visible to all other players. [*Opaque treatment*: The own threshold is visible to each player.]

Part B: Tasks

If you obtained enough Points to reach your threshold **you can earn additional Points in this part by solving various tasks**. To do so you have a time limit of two minutes. For each correctly solved task, you receive 0.8 Points. The Points you earn in this part are added to the Points you obtained in Part A. The tasks are randomly selected and consist of math tasks, trivia questions and similar tasks. At the end of each period, you will be informed about the number of Points you earned by solving the tasks.

If you did not reach your threshold, you cannot earn additional Points, but you can still participate in the tasks. In this case, you earn the Points you obtained in Part A.

- **Example 1**: Your threshold to participate in Part B is 5 Points. The agreement in the communication phase pays you 8 Points. Since you reached your threshold, you can earn additional Points in Part B. Your payoff consists of the 8 Points you already obtained in Part A and the Points which you earn in Part B.

- **Example 2**: Your threshold to participate in Part B is 5 Points. The agreement in the communication phase pays you 4 Points. You cannot earn additional Points in Part B since you did not reach the threshold that was assigned to you. Your payoff in this period consists of the 4 Points you obtained in Part A.

Payoff of Experiment 2

Only one of the seven periods is relevant for your final payoff. At the end of the experiment, **one period will be randomly drawn**. The Points you earned in this period will be paid in Euros.

At the beginning of Experiment 2, we ask you to anser some comprehension questions and to rank distributions according to your preferences. Afterwards, all participants will complete a trial period in order to familiarize yourself with the procedure. Neither the answers of the comprehension questions, nor the ranking of the distributions, nor the outcome of the trial period are relevant for your final payoff.

End

The Points you earned in Experiment 1 and Experiment 2 will be added and converted at the exchange rate 1:1,1 into Euros. This amount will be paid to you at the end of the experiment.

Before the payout, we ask you to answer a questionnaire. Please take your time to answer the questionnaire. You will earn another **4 Points** for a thorough and complete **questionnaire**.

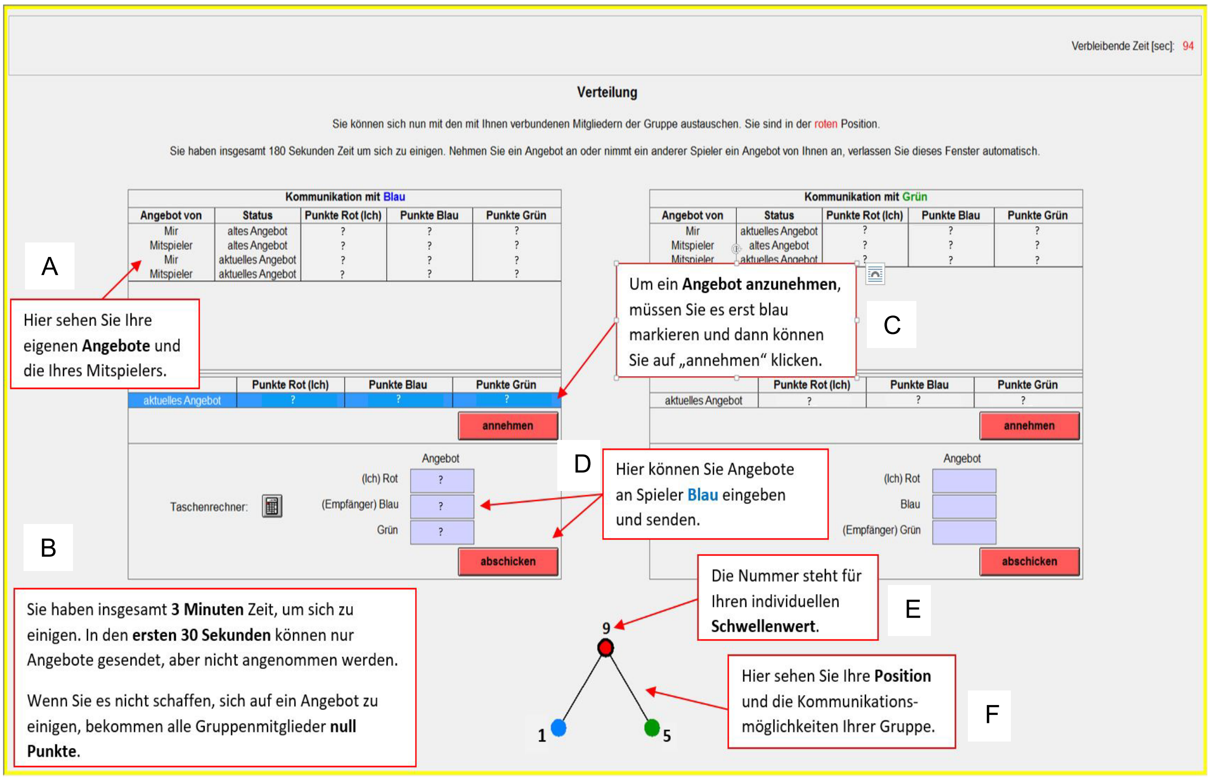

[*Opaque treatment*]

Translations

A: Here you see your own offers and the offers of the other players.

B: You have **3 minutes** to find an agreement. In the **first 30 seconds**, you can only send but not accept offers. If you do not manage to find an agreement, none of the group members receive **any Points**.

C: To **accept an offer**, you must select the offer (it turns blue) and click on "accept".

D: Here you can make and send offers to the **blue** player.

E: The number represents your individual **threshold**.

F: Here you see you position and the exchange channels in your group.

Table A1. Demographic statistics of subjects in the sample

	Experiment 1	Experiment 2	Total
**Location**	Vienna, Austria	Hamburg, Germany	
**Participants**	192	192	384
**Sex**			
Female	108 (56.3%)	112 (58.3%)	220 (57.3%)
Male	84 (43.7%)	80 (41.7%)	164 (42.7%)
**Age (years)**			
Mean (SD)	23.3 (5.6)	25.6 (5.4)	24.4 (5.6)
Median [Min, Max]	22 [17, 57]	24.5 [18, 58]	23 [17, 58]
**Semester**			
Mean (SD)	5.0 (4.5)	6.9 (4.3)	6.0 (4.5)
Median [Min, Max]	3.5 [1, 22]	7 [1, 22]	5 [1, 22]
**Field of Study**			
Natural Sciences	50 (26.0%)	27 (14.1%)	77 (20.1%)
Technical Studies	27 (14.1%)	6 (3.1%)	33 (8.6%)
Economics	22 (11.5%)	63 (32.8%)	85 (22.1%)
Social Sciences	47 (24.5%)	47 (24.5%)	94 (24.5%)
Human Sciences	29 (15.1%)	43 (22.4%)	72 (18.8%)
Life Sciences	17 (8.9%)	6 (3.1%)	23 (6.0%)
**Experimental Experience (prev. participations)**			
Mean (SD)	2.9 (3.7)	13.2 (13.9)	8.1 (11.4)
Median [Min, Max]	2 [0, 30]	10 [0, 100]	4 [0, 100]
**Social value orientation (degree)**			
Mean (SD)	24.1 (14.3)	16.2 (13.2)	20.1 (14.4)
Median [Min, Max]	28.3 [-16.3, 57.8]	7.8 [-16.3, 53.4]	18.4 [-16.3, 57.8]
**Support for the Need Principle (BSJO)**			
Mean (SD)	1.5 (0.7)	1.5 (0.6)	1.5 (0.6)
Median [Min, Max]	1.3 [1, 5]	1.3 [1, 4.7]	1.3 [1, 5]

Table A2. Experiment 1 (Vienna) - Need satisfaction across scenarios and positions

	Need satisfaction												
	Thresholds				Transparent treatment				Opaque treatment				
Period	A	B	C	∑	A	B	C	Ø	A	B	C	Ø	N
1.	0	5	9	14	1.0	1.0	.69	.22	1.0	1.0	.38	.03	32
2.	9	1	5	15	.66	1.0	.78	.31	.53	1.0	.78	.34	32
3.	1	5	12	18	.78	1.0	.56	.09	.81	1.0	.25	.03	32
4.	5	9	1	15	.69	1.0	.88	.56	.94	.84	.84	.66	32
5.	0	0	0	0	1.0	1.0	1.0	1.0	1.0	1.0	1.0	1.0	32
6.	9	5	1	15	.59	1.0	.94	.53	.47	1.0	.84	.31	32
7.	5	5	5	15	.81	1.0	.69	.50	.75	1.0	.81	.56	32
Avg.	3	4.3	4.7	13	.79	1.0	.79	.46	.79	.98	.70	.42	32

Note: In the experiment participated 96 subjects per treatment which equals 32 observations per scenario.

Table A3. Experiment 2 (Hamburg) - Need satisfaction across scenarios and positions

	Need satisfaction												
	Thresholds				Transparent treatment				Opaque treatment				
Period	A	B	C	∑	A	B	C	Ø	A	B	C	Ø	N
1.	1	5	1	7	.94	1.0	.84	.78	.88	1.0	.97	.84	32
2.	9	5	5	19	.75	1.0	.84	.53	.34	1.0	.81	.22	32
3.	5	5	12	22	.72	1.0	.66	.16	.75	1.0	.28	.00	32
4.	5	5	1	11	.81	1.0	.88	.69	.81	1.0	.84	.75	32
5.	12	5	12	29	.5	.97	.38	.00	.32	1.0	.42	.00	32
6.	9	5	9	23	.61	1.0	.61	.29	.41	1.0	.53	.03	32
7.	5	5	5	15	.78	1.0	.66	.44	.72	1.0	.78	.50	32
Avg.	6.6	5	6.4	18	.73	.99	.70	.41	.61	1.0	.66	.34	32

Note: In the experiment participated 96 subjects per treatment which equals 32 observations per scenario.

Table A4. Experiment 1 (Vienna) - Payoffs across scenarios and positions

	Payoffs												
	Thresholds				Transparent treatment				Opaque treatment				
Period	A	B	C	∑	A	B	C	Ø	A	B	C	Ø	N
1.	0	5	9	14	5.3	9.9	8.8	8	6.6	9.6	7.8	8	32
2.	9	1	5	15	7.3	10.0	6.7	8	8.3	9.2	6.5	8	32
3.	1	5	12	18	5.8	10.3	7.9	8	6.6	10.1	7.3	8	32
4.	5	9	1	15	6.2	12.2	5.6	8	7.8	9.8	6.4	8	32
5.	0	0	0	0	7.3	10.6	6.1	8	6.6	9.8	7.6	8	32
6.	9	5	1	15	6.4	11.1	6.5	8	7.7	10.3	6.0	8	32
7.	5	5	5	15	7.0	11.1	5.9	8	6.4	10.5	7.1	8	32
Avg.	3	4.3	4.7	13	6.5	10.8	6.8	8	7.1	9.9	7.0	8	32

Note: In the experiment participated 96 subjects per treatment which equals 32 observations per scenario.

Table A5. Experiment 2 (Hamburg) - Payoffs across scenarios and positions

	Payoffs												
	Thresholds				Transparent treatment				Opaque treatment				
Period	A	B	C	∑	A	B	C	Ø	A	B	C	Ø	N
1.	1	5	1	7	7.8	9.9	6.3	8	7.1	9.1	7.8	8	32
2.	9	5	5	19	8	9.6	6.4	8	7.2	9.7	7.2	8	32
3.	5	5	12	22	5.6	9.3	9.1	8	7.1	9.9	7.0	8	32
4.	5	5	1	11	7.0	10.4	6.7	8	7.1	9.9	6.9	8	32
5.	12	5	12	29	7.3	10.3	6.4	8	6.2	11.0	6.8	8	32
6.	9	5	9	23	7.1	9.9	7.0	8	6.5	10.7	6.8	8	32
7.	5	5	5	15	7.5	10.5	6.0	8	6.6	10.3	7.1	8	32
Avg.	3	4.3	4.7	13	7.2	10.0	6.8	8	6.8	10.1	7.1	8	32

Note: In the experiment participated 96 subjects per treatment which equals 32 observations per scenario.

Table A6. Experiment 1 (Vienna) – Dyad members across scenarios and positions

	Dyad member												
	Thresholds				Transparent treatment				Opaque treatment				
Period	A	B	C	∑	A	B	C	Ø	A	B	C	Ø	N
1.	0	5	9	14	.53	1.0	.47	.67	.66	1.0	.34	.67	32
2.	9	1	5	15	.56	1.0	.44	.67	.53	1.0	.47	.67	32
3.	1	5	12	18	.50	1.0	.50	.67	.69	1.0	.31	.67	32
4.	5	9	1	15	.44	1.0	.56	.67	.56	1.0	.44	.67	32
5.	0	0	0	0	.50	1.0	.50	.67	.47	1.0	.53	.67	32
6.	9	5	1	15	.56	1.0	.44	.67	.50	1.0	.50	.67	32
7.	5	5	5	15	.63	1.0	.38	.67	.47	1.0	.53	.67	32
Avg.	3	4.3	4.7	13	.53	1.0	.47	.67	.55	1.0	.45	.67	32

Note: In the experiment participated 96 subjects per treatment which equals 32 observations per scenario.

Table A7. Experiment 2 (Hamburg) – Dyad members across scenarios and positions

	Dyad member												
	Thresholds				Transparent treatment				Opaque treatment				
Period	A	B	C	∑	A	B	C	Ø	A	B	C	Ø	N
1.	1	5	1	7	.53	1.0	.47	.67	.59	1.0	.41	.67	32
2.	9	5	5	19	.66	1.0	.34	.67	.44	1.0	.56	.67	32
3.	5	5	12	22	.47	1.0	.53	.67	.69	1.0	.31	.67	32
4.	5	5	1	11	.53	1.0	.47	.67	.56	1.0	.44	.67	32
5.	12	5	12	29	.56	1.0	.44	.67	.45	1.0	.55	.67	32
6.	9	5	9	23	.52	1.0	.48	.67	.41	1.0	.59	.67	32
7.	5	5	5	15	.69	1.0	.31	.67	.47	1.0	.53	.67	32
Avg.	3	4.3	4.7	13	.57	1.0	.44	.67	.52	1.0	.48	.67	32

Note: In the experiment participated 96 subjects per treatment which equals 32 observations per scenario.

Table A8. Determinants of outsider's need satisfaction

	DV: Need satisfaction of the third network member				
	I	II	III	IV	V
Opaque need thresholds (ref. = transparent)	-0.582***	-0.579**	-0.578**	-0.579**	-0.596**
	(0.223)	(0.245)	(0.245)	(0.245)	(0.246)
Low need thresholds (ref. = Moderate)	-1.063***	-1.065***	-1.049***	-1.118***	-0.690**
	(0.200)	(0.220)	(0.216)	(0.227)	(0.271)
High need thresholds	1.209***	1.372***	1.429***	1.366***	0.847***
	(0.200)	(0.192)	(0.228)	(0.235)	(0.220)
SVO of the strong dyad member		0.040***	0.040***	0.042***	0.043***
		(0.009)	(0.009)	(0.009)	(0.010)
SVO of the weak dyad member		0.005	0.006	0.009	0.009
		(0.008)	(0.008)	(0.007)	(0.007)
SVO of the outsider		-0.012*	-0.012*	-0.010	-0.009
		(0.007)	(0.007)	(0.008)	(0.008)
Period			0.038	0.038	0.039
			(0.050)	(0.051)	(0.051)
Location: Vienna (ref. = Hamburg)				-0.275	-0.305
				(0.271)	(0.281)
Opaque x Low need thresholds					-1.290***
					(0.425)
Opaque x High need thresholds					1.030***
					(0.380)
Constant	0.065	-0.714**	-0.849**	-0.821**	-0.843**
	(0.166)	(0.356)	(0.379)	(0.392)	(0.381)
Observations	741	741	741	741	741
AIC	913.963	869.785	870.869	870.913	855.497
BIC	932.395	902.041	907.733	912.385	906.185
Log Likelihood	-452.981	-427.892	-427.434	-426.456	-416.748

Logit model with clustered s.e. on the independent group level. *** p < .01, ** p < .05, * p < .10.
Low need thresholds include the combinations 1-5-1, 5-5-1, 5-9-1;
Moderate need thresholds include the combinations 5-5-5, 9-5-0, 9-5-1, 9-1-5, 9-5-5;
High need thresholds include the combinations 1-5-1, 5-5-1, 5-9-1; where the number in the center and sides represent the need threshold of the strong and weak network member, respectively (see, Table 1).

Table A9. Determinants of outsider's need satisfaction

	DV: Need satisfaction of the third network member		
	I	II	III
Opaque need thresholds (ref. = transparent)	-0.122***	-0.114**	0.069
	(0.047)	(0.048)	(0.100)
Thresholds: 1-5-1 (ref. = 5-5-5)	0.172***	0.191***	0.244***
	(0.067)	(0.065)	(0.091)
Thresholds: 5-5-1	0.313***	0.334***	0.341***
	(0.063)	(0.061)	(0.073)
Thresholds: 5-9-1	0.188***	0.164**	0.095
	(0.069)	(0.069)	(0.078)
Thresholds: 9-5-0	-0.283***	-0.327***	-0.098
	(0.085)	(0.084)	(0.119)
Thresholds: 9-5-1	-0.031	-0.064	0.051
	(0.076)	(0.077)	(0.103)
Thresholds: 9-1-5	-0.047	-0.079	0.025
	(0.072)	(0.070)	(0.071)
Thresholds: 9-5-5	-0.062	-0.037	0.186**
	(0.089)	(0.093)	(0.095)
Thresholds: 9-5-9	-0.340***	-0.318***	-0.155
	(0.073)	(0.076)	(0.097)
Thresholds: 12-5-1	-0.234***	-0.261***	-0.133
	(0.078)	(0.078)	(0.124)
Thresholds: 12-5-5	-0.266***	-0.241***	-0.033
	(0.069)	(0.073)	(0.071)
SVO of the strong dyad member		0.008***	0.008***
		(0.002)	(0.002)
SVO of the weak dyad member		0.002	0.002
		(0.001)	(0.001)
SVO of the outsider		-0.002	-0.002
		(0.002)	(0.001)
Opaque x Thresholds: 1-5-1			-0.104
			(0.133)
Opaque x Thresholds: 5-5-1			-0.013
			(0.115)
Opaque x Thresholds: 5-9-1			0.137
			(0.124)
Opaque x Thresholds: 9-5-0			-0.432***
			(0.156)
Opaque x Thresholds: 9-5-1			-0.228
			(0.150)
Opaque x Thresholds: 9-1-5			-0.206
			(0.140)
Opaque x Thresholds: 9-5-5			-0.445***
			(0.155)
Opaque x Thresholds: 9-5-9			-0.324**
			(0.132)
Opaque x Thresholds: 12-5-1			-0.256*
			(0.150)
Opaque x Thresholds: 12-5-5			-0.416***
			(0.110)
Constant	0.561***	0.385***	0.290***
	(0.055)	(0.084)	(0.078)
Observations	741	741	741
Adjusted R^2	0.155	0.213	0.237

Linear probability model with clustered s.e. on the independent group level. *** p < .01, ** p < .05, * p < .10.

Table A10. Determinants of outsider's payoff

	DV: Payoff of the third network member		
	I	II	III
Opaque need thresholds (ref. = transparent)	0.701	0.793*	0.658
	(0.473)	(0.473)	(0.744)
Thresholds: 0-0-0 (ref. = 5-5-5)	0.891*	0.505	-0.251
	(0.481)	(0.484)	(0.648)
Thresholds: 1-5-1	0.687	1.022**	1.026
	(0.420)	(0.434)	(0.694)
Thresholds: 5-5-1	1.625***	1.986***	2.070***
	(0.470)	(0.448)	(0.650)
Thresholds: 5-9-1	0.359	-0.021	-0.737
	(0.435)	(0.437)	(0.550)
Thresholds: 9-5-0	0.953**	0.534	0.425
	(0.447)	(0.426)	(0.583)
Thresholds: 9-5-1	-0.359	-0.833*	-1.545**
	(0.430)	(0.471)	(0.644)
Thresholds: 9-1-5	0.906**	0.440	0.485
	(0.427)	(0.413)	(0.532)
Thresholds: 9-5-5	1.125**	1.524***	1.892***
	(0.457)	(0.452)	(0.644)
Thresholds: 9-5-9	-0.481	-0.102	0.307
	(0.454)	(0.459)	(0.750)
Thresholds: 12-5-1	-0.109	-0.520	-1.160
	(0.558)	(0.559)	(0.858)
Thresholds: 12-5-5	0.141	0.530	1.142*
	(0.467)	(0.490)	(0.643)
Thresholds: 12-5-12	-2.073***	-1.758***	-1.299**
	(0.473)	(0.505)	(0.631)
SVO of the strong dyad member		0.076***	0.077***
		(0.014)	(0.015)
SVO of the weak dyad member		0.037***	0.038***
		(0.013)	(0.012)
SVO of the outsider		-0.001	-0.001
		(0.012)	(0.011)
Opaque x Thresholds: 0-0-0			1.501
			(0.918)
Opaque x Thresholds: 1-5-1			0.002
			(0.906)
Opaque x Thresholds: 5-5-1			-0.157
			(0.914)
Opaque x Thresholds: 5-9-1			1.421*
			(0.805)
Opaque x Thresholds: 9-5-0			0.207
			(0.856)
Opaque x Thresholds: 9-5-1			1.414
			(0.902)
Opaque x Thresholds: 9-1-5			-0.101
			(0.838)
Opaque x Thresholds: 9-5-5			-0.723
			(0.928)
Opaque x Thresholds: 9-5-9			-0.793
			(0.901)
Opaque x Thresholds: 12-5-1			1.270
			(1.102)
Opaque x Thresholds: 12-5-5			-1.213
			(0.958)
Opaque x Thresholds: 12-5-12			-0.923
			(0.929)
Constant	3.696***	1.349**	1.379**
	(0.382)	(0.599)	(0.631)
Observations	894	894	894
Adjusted R^2	0.053	0.161	0.165

Maximum likelihood model with clustered s.e. on independent group level. *** p < .01, ** p < .05, * p < .10.
